# Dezocine Prevents Postoperative Hyperalgesia in Patients Undergoing Open Abdominal Surgery

**DOI:** 10.1155/2015/946194

**Published:** 2015-06-11

**Authors:** Fang Yu, Jie Zhou, Suyun Xia, Huan Xu, Xiangrui Wang

**Affiliations:** Department of Anesthesiology, Renji Hospital, School of Medicine, Shanghai Jiao Tong University, Shanghai 200127, China

## Abstract

*Objective*. Postoperative hyperalgesia is very frequent and hard to treat. Dezocine is widely used and has a modulatory effect for thermal hyperalgesia in animal models. So, this study was designed to investigate the potential role of dezocine in decreasing postoperative hyperalgesia for patients undergoing open abdominal surgery. *Methods*. This is a randomized, double-blinded, and placebo-controlled trial. 50 patients for elective open gastrectomy were randomly allocated to either a true treatment group (0.15 mg/kg intravenous dezocine at the end of surgery) or a sham treatment group (equivalent volume of saline) in a 1 : 1 ratio. Patients were followed up for 48 hours postoperatively and pain threshold to Von Frey filaments, pain scores, PCIA consumption, rescue analgesics use, sedation score, and occurrence of postoperative nausea and vomiting were recorded. *Results*. Patients in the true treatment group experienced statistically significantly higher pain threshold on forearm and smaller extent of peri-incisional hyperalgesia than the sham treatment group. Rescue analgesic use, cumulative PCIA consumption, and pain scores were statistically significantly decreased in the true treatment group compared to the sham treatment group. *Conclusions*. Dezocine offers a significant antihyperalgesic and analgesic effect in patients undergoing elective open gastrectomy for up to 48 hours postoperatively.

## 1. Introduction

Hyperalgesia frequently occurs after surgery, especially in the early postoperative period [1]. The occurrence of postoperative hyperalgesia can be either due to nervous system sensitization by surgical nociception or as an adverse effect of perioperative opioid use. Nociception induced hyperalgesia is generally considered as a consequence of surgical tissue and nerve trauma [2] while opioid induced hyperalgesia (OIH) is a paradoxical response whereby a patient receiving opioids during surgery for the treatment of pain may actually become more sensitive to certain painful stimuli after surgery [3]. Postoperative hyperalgesia may occur in a restricted area where the pain is treated or in a more generalized manner [4].

Remifentanil is a potent *μ* opioid receptor agonist. Its short context sensitive half-life and short elimination half time make it suitable for anesthetic maintenance during surgery. After intravenous administration, remifentanil exhibits a stable plasma concentration [5], organ independent elimination [6, 7], minimal alveolar concentration reducing effect [8], and attenuated autonomic, somatic, and adrenocortical responses to noxious stimuli [9]. Nevertheless, remifentanil-induced hyperalgesia (RIH) is more severe and frequent than other opioids [10, 11]. Postoperative hyperalgesia is always complicated with RIH and other contributing factors including tissue trauma, whereas the precise mechanisms remain unclear [12]. Postoperative hyperalgesia usually results in unsatisfied pain control and increased extra morphine consumption. Current treatments such as opioids, ketamine, and NSAIDs drugs are insufficient to solve this problem. So, novel strategies are highly needed.

Dezocine, first developed in 1970s, is an opioid *μ* receptor partial agonist/antagonist [13]. Dezocine is largely preferred as an alternative medication for perioperative pain management because of its good tolerance, mild adverse effects, and good potence [14]. There has always been debate for its *κ* receptor profile. Although initially identified as a *κ* receptor agonist, recent studies suggest that dezocine might be a *κ* receptor antagonist [13, 15]. As *κ* receptor antagonist, it can probably have a modulatory effect on spinal dynorphin, preventing it from binding to *κ* receptor, which plays a crucial role in the spinal effect of hyperalgesia [3]. Studies have also showed that dezocine produced high levels of thermal hyperalgesia modulatory function in animal models [16]. However, whether clinically administrated dezocine could modulate postoperative hyperalgesia and provide a more satisfying pain control has never been documented.

Therefore, the present study aimed at investigating the effect of dezocine in preventing postoperative hyperalgesia using a random double-blinded study.

## 2. Methods

### 2.1. Study Design and Patient Population

This was a double-blinded, placebo-controlled randomized trial. This study was approved by the Ethic Committee of Shanghai Renji Hospital affiliated to Shanghai Jiaotong University of Medicine (document number 2012032) and was also registered in the Chinese Clinical Trials Registry (ChiCTR-TRC-14004723). Patients scheduled to have open gastrectomy from August 2012 to February 2014 were included in this study.

### 2.2. Criteria for Inclusion and Exclusion

Patients were included if they were (1) aged 18~64 years and (2) with American Society Anesthesiologists physical statuses I-II.

Patients were excluded when (1) they had chronic use of analgesics or had used opioids within 24 h of surgery; (2) immediate extubation was not planned after surgery; (3) patients had neurological or psychiatric disorders; (4) there was end-stage cancer in preoperative evaluation; (5) there was diabetes or severe hypertension; (6) there were either abnormal findings in laboratory tests including complete blood count, liver and renal function, electrolytic analysis, and thrombin time; (7) they were unable to understand the usage of the patient-controlled intravenous analgesia (PCIA) device; (8) body mass index (BMI) was > 30 or < 17; (9) there was alcoholic abuse or coffee drinking > 2 cups/day, and (10) there was rejection either by the surgeon or by the patient. Moreover, after initial enrollment, if any of the following activities occurred, the patients were also excluded: (1) ICU hospitalization after surgery; (2) transient perioperative hypothermia; (3) blood loss > 400 mL or hemoglobin decreasing > 30% in the last perioperative ABG; (4) perioperative acid-alkaline or electrolytes disorders; (5) advanced gastric cancer found on the surgical table. After initial inclusion and exclusion, we had 93 patients enrolled in the study and 43 patients were finally excluded because of perioperative findings as listed above.

### 2.3. Preoperative Preparations

During the preoperative evaluation on the day before surgery, after informed consent was signed, patients were instructed in the use of the PCIA pump, the quantitative sensory tests with calibrated Von Frey filaments (0.6–180 g/mm^2^, North Coast Medical, Inc.), the visual analogue scale (VAS; from 0 to 10; 0, no pain; 10, worst pain imaginable), and a four-point verbal rating scale (VRS) for pain evaluation (0, no pain; 1, slight pain; 2, moderate pain; 3, intense or severe pain). The static hyperalgesia was also assessed proximally to the surgical wound and on the forearm. Tactile pain thresholds were measured 2 to 3 cm away from the potential incision at 3 levels (top, middle, and bottom), separated by about 5 cm on the right side. It is defined as the smallest force that was just perceived as painful [17]. No premedication or fasting was required after midnight on the day before surgery.

### 2.4. Anesthesia Protocol

In the operating room, standard monitoring was performed and the baseline values were recorded. Anesthesia was induced with midazolam 0.05 mg/kg, propofol 1.5 mg/kg, fentanyl 4 *μ*g/kg, and rocuronium 0.6 mg/kg to facilitate the tracheal intubation. After tracheal intubation, the patients were ventilated to normocapnia. An infusion of a cisatracurium was reached at 2 *μ*g/kg/min and was discontinued about 15 min before the end of the surgery. Residual neuromuscular blockade was antagonised by 15–20 *μ*g/kg atropine and 40–60 *μ*g/kg neostigmine. Anesthesia was maintained with remifentanil at 0.4 *μ*g/kg/min and sevoflurane at a concentration from 1% to 2.5%.

### 2.5. Sample Size Estimation

In our preliminary of 20 patients, we concluded that an estimated sample size of 22 patients per group would give us a *β*-risk of 80% at an *α*-level of 0.05 for detecting a difference of 30% in the PCIA consumption. We have also anticipated a lost prevalence of 10%. Thus, the study size was prospectively set to 50 patients (25 patients/group).

### 2.6. Assignment and Postoperative Management

These 50 patients were randomly assigned to one of the two groups: dezocine group and control group. Each group had a random-number generated by computer precising the group assignment and envelopes containing the results were prepared. In the morning of surgery, a person not involved in the evaluation procedure opened the envelope and prepared the drugs. Thirty minutes before the end of the surgery, a titration of 0.1 *μ*g/kg sufentanil was given intravenously for postoperative analgesia. After incision closure, sevoflurane and remifentanil were discontinued, a dose of 0.15 mg/kg dezocine (Yangtze River Pharmaceutical Group, Taizhou, Jiangsu, China) was administered to dezocine group, and the same volume of normal saline was administered to group control. The patients and the investigators involved in patient management or data collection were all unaware of the group assignment.

Inspired sevoflurane concentration was increased stepwise by 0.5%–1% when insufficient anesthesia was considered which was defined as an accelerated heart rate by 15% or a systolic arterial blood pressure exceeding baseline values by 20% with or without clinical signs of inadequate anesthesia such as patient movement, coughing, and tearing. Inadequate anesthesia was also treated with propofol. Atropine and ephedrine were prepared to treat bradycardia and hypotension perioperatively. After recovery of adequate spontaneous ventilation and the obeisance to verbal commands (eye opening and limb moving), the tracheal tube was removed and the patients were kept in the postanesthesia care unit (PACU) for an hour, where standard monitoring was recorded every 15 min. Additional analgesics were given to the patients who had VAS > 5. Background infusion of PCIA was started just before leaving PACU and the postoperative pain was controlled by PCIA, which was programmed to deliver demand doses of sufentanil 3*μ*g/h with a 15 min lockout and continuous infusion of 3*μ*g/h. In general ward this PCIA regimen was maintained and rescue analgesics were administered if patient required extra pain control or VAS > 5.

### 2.7. Measurements

Baseline MAP and HR were defined as the mean of the two lowest measurements recorded during a 5 min interval just before the induction of anesthesia. Values from all routine anesthetic monitors were recorded at a 5 min interval perioperatively. Duration of anesthesia and surgery, the total doses of remifentanil, and the use of atropine or ephedrine were also recorded.

Rescue analgesics, VAS score evaluating the pain intensity, and sedation score monitored by Ramsay scale (1, anxious and agitated; 2, cooperative, tranquil, and oriented; 3, responding only to verbal commands; 4, asleep with brisk response to light stimulation; 5, asleep without response to light stimulation; 6, nonresponsive) were recorded during the PACU stay. The cumulative consumption of sufentanil given by PCIA and pain scores (VAS and VRS) were also recorded at 6, 24, and 48 h after surgery. The primary outcome was the consumption of sufentanil during the first 24 h after surgery. The incidences of postoperative nausea and vomiting were recorded during the visit at 48 h postoperatively. Subjects who experienced severe postoperative nausea and vomiting (PONV) were treated with ondansetron 4 mg intravenously. Other side effects such as respiratory depression, muscle rigidity, pruritus, and dysphoria were also recorded.

The pain threshold for mechanical static stimuli was evaluated both at 2 cm proximal to the surgical wound and on the forearm by calibrated Von Frey filaments (0.6–180 g/mm^2^) at 6, 24, and 48 h after surgery. The extent of mechanical static hyperalgesia to punctuate stimulation proximal to the wound was assessed with Von Frey filament number 17 (60 g/mm^2^) as previously described [17]. Hyperalgesia was determined by stimulating along three linear paths at right angles to the top, middle, and bottom side of the surgical wound in steps of 0.5 cm at 1 s interval, starting from 10 cm outside the surgical wound. The distance (in cm) from the incision to where sensations changed was measured and a total of the three measurements were calculated and used for statistical comparisons.

### 2.8. Statistical Analysis

Age, weight, height, BMI, duration of surgery and anesthesia, intraoperative remifentanil use, haemodynamic variables, cumulative sufentanil consumption, and VAS scale were analysed by Student's *t*-test. The *χ* ^2^ test was used to compare the sex, intraoperative atropine or ephedrine use, PONV incidence, and PACU rescue analgesics use. Mann-Whitney test was applied to compare the pain threshold, the extent of mechanical static hyperalgesia, VRS scores, and Ramsay scale during PACU stay. All data analysis was performed using SPSS (version 13.0, IBM). *P* values less than 0.05 were considered to be statistical significance.

## 3. Results

A total of 50 patients (25 patients in each group) were recruited in our study.  Table 1 shows the patients' morphometric and demographic characteristics as well as the details of surgery. No significant difference was found between the two groups (*P* > 0.05).

**Figure 1:**
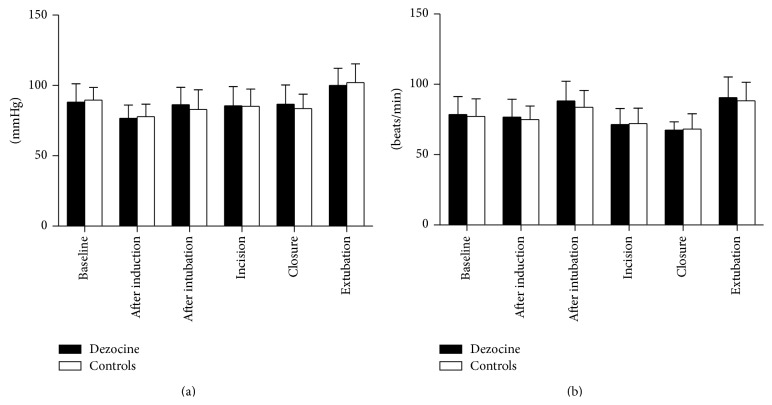
Mean arterial pressure (MAP) (a) and heart rate (HR) (b) (mean ± SD) values at various times points. No statistical significant difference was observed.

During the surgical procedure, hemodynamic status and drug consumption were noted.  Table 2 presents the perioperative drug consumption including remifentanil, atropine, and ephedrine, and no significant difference was observed between the two groups (*P* > 0.05).  Figure 1 shows perioperative mean arterial pressure (MAP) and heart rate (HR), which were comparable between the two groups (*P* > 0.05).

**Figure 2:**
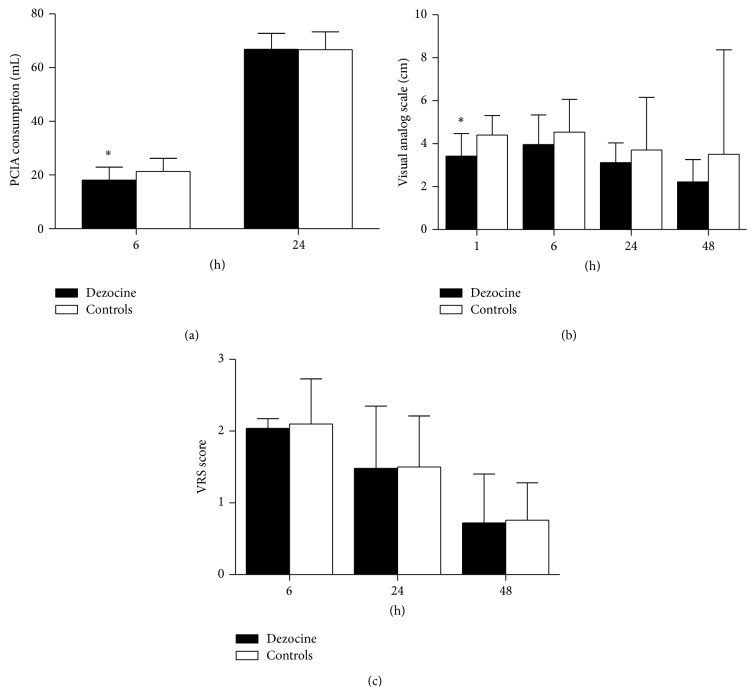
Postoperative PCIA consumption (a), VAS score (b), and VRS score (c) (mean ± SD). ^∗^ *P* < 0.05.

**Figure 3:**
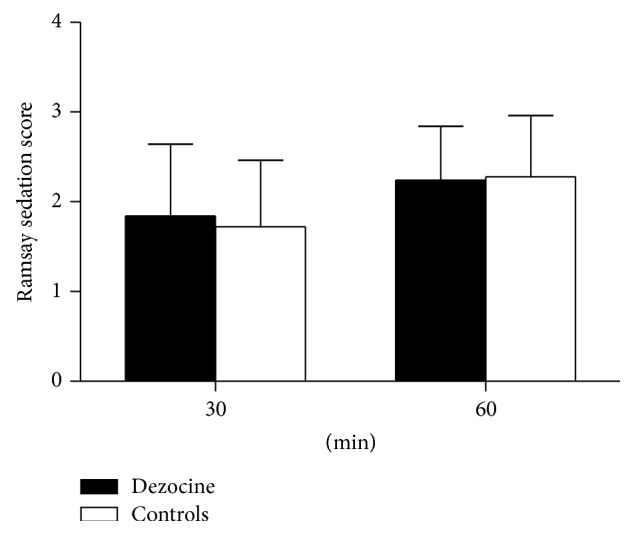
Ramsay sedation score during PACU stay (mean ± SD).

**Figure 4:**
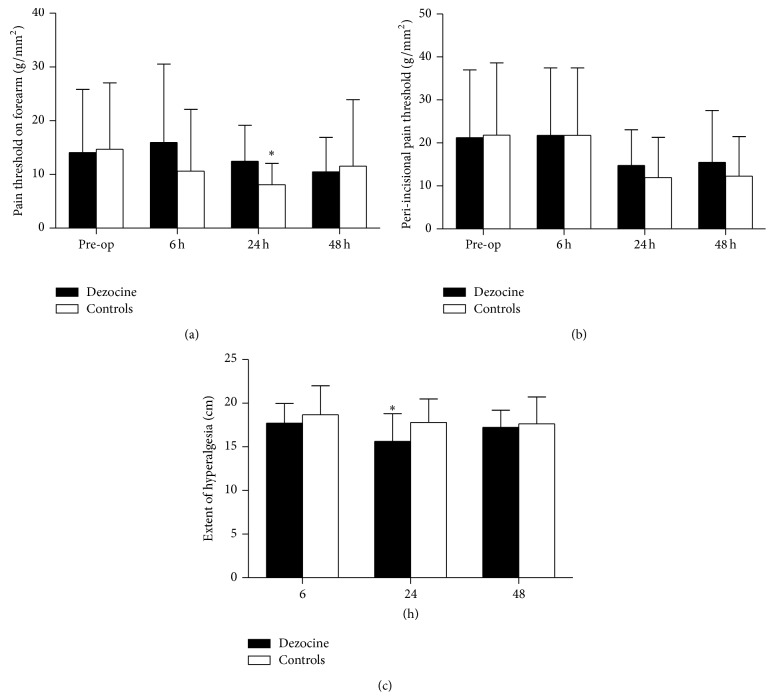
Pain threshold on forearm (a) and proximal to the surgical wound (b) and the extent of peri-incisional hyperalgesia (mean ± SD). ^∗^ *P* < 0.05.

During postoperative follow-up, patients treated with dezocine showed statistically significantly decreased PCIA consumption at 6 postoperative hours (*P* < 0.05) and lower VAS pain score at the first hour after the surgery compared with the patients in the control group (*P* < 0.05) (Figure 2).  Table 3 shows that patients in the treatment group required statistically significantly less rescue analgesic during PACU stay (*P* < 0.05).  Figure 4 showed that, at 24 postoperative hours, pain threshold on forearm of the patients in the treatment group was statistically significantly higher than the patients in the control group (*P* < 0.05). The extent of peri-incisional hyperalgesia was statistically significantly smaller in patients of the treatment group than the control group (*P* < 0.05) (Figure 4), whereas no significant difference was observed in postoperative VRS pain score, peri-incisional pain threshold, incidence of postoperative hyperalgesia, Ramsay sedation score (Figure 3), and the incidence of PONV (*P* > 0.05).

## 4. Discussion

To the best of our knowledge, this study firstly showed that intravenous injection of 0.15 mg/kg dezocine at the end of surgery leads to a better postoperative pain control manifesting as indicated by a fewer PACU rescue analgesics demands, a decreased cumulative postoperative PCIA consumption during the first 6 postoperative hours, and a more satisfactory VAS score at 1 hour postoperatively. Dezocine can also provide antihyperalgesia effects, including a higher pain threshold on the forearm and a smaller extent of hyperalgesia proximal to the surgical wound.

Pain after surgery is a major management challenge in clinical practice and postoperative hyperalgesia is usually complicated in such situations. A considerable number of surgical patients suffer from moderate to severe acute postoperative pain and a large number of patients experience inadequate pain relief with available pain managements. Postoperative hyperalgesia manifesting as an exaggerated original underlying painful condition or a more generalized state mainly involves both surgical nociception and opioid administrations and it is considered as a very important contributive cause of inadequate postoperative pain control [3]. Postoperative hyperalgesia usually results in higher postoperative pain scores and earlier morphine consumption requirements, thus leading to an increased occurrence of adverse effects in patients [17, 18], making it a clinical management concern. Although being studied for several years, the exact molecular mechanism seems to be multifactorial and is not clear yet. But the possible mechanisms of postoperative hyperalgesia are thought to share similar underlying mechanisms such as the involvement of the sensitization of primary neurons, neuroplastic changes of central pain transmission pathway [3], and excitatory amino acids via the N-methyl-D-aspartate (NMDA) receptor [1]. Our study applying Von Frey filaments has showed decreased pain threshold in dezocine and control groups, proving the evidence of the existence of postoperative hyperalgesia. We did an infusion of remifentanil at an initial dose of 0.4 *μ*g/kg/min and then adjusted to the hemodynamic changes or the anesthetic depth for the maintenance of anesthesia, which is widely considered dose-dependently [17] to generate remifentanil-induced hyperalgesia [19]. Surgical nociception and opioid induced hyperalgesia share the same molecular mechanism including sensitization of primary afferent neurons, enhanced production and release of excitatory neurotransmitters, sensitization of second-order neurons to excitatory neurotransmitters, and neuroplastic changes in the spinal level which lead to upregulation of spinal dynorphin. It should be pointed out that postoperative hyperalgesia can be a more generalized state. We considered the hyperalgesic state on the forearm as a generalized state and our results showed that high incidence of generalized postoperative hyperalgesia is observed in our cohort, which was accordant with other reports [20]. Here based on a random double-blinded study, dezocine is found to be effective in preventing postoperative hyperalgesia in patients undergoing open abdominal surgery.

Dezocine, as a combined opioid agonist/antagonist, is of great interest because of its ability to produce analgesia, decreased liability to addiction, and limited depression of respiration compared with the classical opioid [21]. Clinical observations have suggested that analgesic efficacy of dezocine was similar to that of morphine [22] and a combined use of dezocine with morphine can greatly increase the analgesic effects, which indicates that dezocine may have an additional mechanism of analgesic effect [21]. This is because dezocine should diminish the analgesia effect significantly as a partial *μ* receptor antagonist. A recently published study identified that dezocine is a *κ* opioid antagonist and also inhibits norepinephrine and serotonin reuptake in vitro [13], which may have potential interactions with antinociceptive pathways since antagonistic effect of dezocine against spinal dynorphin might play a nonnegligible role in the central pathway of nociceptive transmission. Here we reported that dezocine could greatly increase the generalized pain threshold instead of relieving the peri-incisional hyperalgesia, which might explain the central modulatory effect and further studies should be carried out to explain the exact molecular mechanism.

Several limitations of the present study should be highlighted. Firstly, the dosage of remifentanil used in our study was less than 0.4 *μ*g/kg/min and 0.4 *μ*g/kg/min is the most widely reported concentration inducing significant postoperative RIH in the literature. In the clinic, we found that patients with high perioperative dose of remifentanil (0.4 *μ*g/kg/min) had frequently unstable haemodynamics and usually required repeated administration of ephedrine and atropine. Thus, in terms of patients' safety and as required by both of the anesthesiologists and the surgeons, we decreased the dose and hyperalgesia was still induced as proven by the quantitative pain test, calibrated Von Frey filaments. Secondly, we did not note the delay of the first demand of PACU rescue analgesics and the dosage of the analgesics used during PACU stay. The length of PACU stay was not assumed, which should be dependent on the patient status. But in our institute it might be more dependent on the department policy which usually requires one-hour PACU stay. Thirdly, our study was powered on the postoperative PCIA consumption. A larger study powered on the pain threshold change for about several weeks warrants being conducted to see the long term hyperalgesia relief effects of dezocine since evidences have showed that there might be a link between postoperative hyperalgesia and the risk of chronic pain development.

In conclusion, our study shows that, for patients undergoing open gastrectomy, intravenous injection of dezocine at the end of surgery decreases PCIA consumption and helps achieve a better postoperative pain control as well as a decreased generalized and peri-incisional extent of hyperalgesia level. Dezocine may be a potentially useful adjunctive agent for the management of postoperative hyperalgesia and further studies are needed to investigate the optimal dose and to explain the mechanisms.

## 5. Conclusions

This prospective, double-blinded, and randomised study has confirmed that dezocine provides analgesic and antihyperalgesic effects for patients undergoing elective open gastrectomy. No adverse effects were observed for up to 48 postoperative hours.

## Figures and Tables

**Table 1:** Morphometric and demographic data and details of surgery.

	Dezocine	Controls	*P* value
Sex (F/M)	9/16	7/18	0.77
Age (yr)	54.64 ± 10.71	54.44 ± 7.27	0.94
Weight (kg)	63.96 ± 9.21	69.48 ± 10.94	0.06
Height (m)	1.68 ± 0.07	1.70 ± 0.08	0.39
BMI (kg/m^2^)	22.62 ± 2.64	23.99 ± 2.85	0.08
Duration of anesthesia (h)	3.75 ± 0.91	4.08 ± 0.80	0.20
Duration of surgery (h)	3.43 ± 0.65	3.29 ± 0.78	0.49
Gastric cancer/gastric ulcer	22/3	24/1	0.30
Partial gastrectomy/total gastrectomy	10/15	8/17	0.56

All values, except sex, diagnosis, and surgical type, are expressed as mean ± SD.

**Table 2:** Perioperative drug consumption.

	Dezocine	Controls	*P* value
Remifentanil (mg)	3.00 ± 0.63	2.85 ± 0.70	0.40
Atropine	8/17	10/15	0.56
Ephedrine	8/17	7/18	0.76

Remifentanil consumption is expressed as mean ± SD; atropine and ephedrine consumption were showed as number of patients using drugs versus number of patients without administration of those drugs.

**Table 3:** Extra analgesic consumption, PONV, and incidence of hyperalgesia.

	Dezocine	Controls	*P* value
Analgesic use in PACU	10/25 (40%)	18/25 (72%)	0.02
PONV	15/25 (60%)	11/25 (44%)	0.26
Peri-incisional hyperalgesia	22/25 (88%)	20/25 (80%)	—
Generalized hyperalgesia	14/25 (56%)	20/25 (80%)	0.07

All data are expressed as number of patients.
